# A pangenome graph reference of 30 chicken genomes allows genotyping of large and complex structural variants

**DOI:** 10.1186/s12915-023-01758-0

**Published:** 2023-11-22

**Authors:** Edward S. Rice, Antton Alberdi, James Alfieri, Giridhar Athrey, Jennifer R. Balacco, Philippe Bardou, Heath Blackmon, Mathieu Charles, Hans H. Cheng, Olivier Fedrigo, Steven R. Fiddaman, Giulio Formenti, Laurent A. F. Frantz, M. Thomas P. Gilbert, Cari J. Hearn, Erich D. Jarvis, Christophe Klopp, Sofia Marcos, Andrew S. Mason, Deborah Velez-Irizarry, Luohao Xu, Wesley C. Warren

**Affiliations:** 1 Bond Life Sciences Center, University of Missouri, Columbia, MO USA; 2 Faculty of Veterinary Medicine, Ludwig-Maximilians-Universität, Munich, Germany; 3 Center for Evolutionary Hologenomics, Globe Institute, University of Copenhagen (UCPH), Copenhagen, Denmark; 4 Department of Ecology & Evolutionary Biology, Texas A&M University, College Station, TX USA; 5 Department of Poultry Science, Texas A&M University, College Station, TX USA; 6 Vertebrate Genome Laboratory, The Rockefeller University, New York, NY USA; 7 Sigenae, GenPhySE, Université de Toulouse, INRAE, ENVT, Castanet Tolosan, 31326 France; 8 Department of Biology, Texas A&M University, College Station, TX USA; 9 University Paris-Saclay, INRAE, AgroParisTech, GABI, Sigenae, Jouy-en-Josas, France; 10 Avian Disease and Oncology Laboratory, USDA, ARS, USNPRC, East Lansing, MI USA; 11 Department of Biology, University of Oxford, Oxford, OX1 3SZ UK; 12 School of Biological and Behavioural Sciences, Queen Mary University of London, London, E1 4DQ UK; 13 The Howard Hughes Medical Institute, Chevy Chase, MD USA; 14 Sigenae, Genotoul Bioinfo, MIAT UR875, INRAE, Castanet Tolosan, France; 15 Applied Genomics and Bioinformatics, University of the Basque Country (UPV/EHU), Leioa, Bilbao, Spain; 16 Department of Biology, The University of York, York, UK; 17 Key Laboratory of Freshwater Fish Reproduction and Development (Ministry of Education), Key Laboratory of Aquatic Science of Chongqing, School of Life Sciences, Southwest University, Chongqing, 400715 China; 18 Department of Animal Sciences, University of Missouri, Columbia, MO USA

**Keywords:** Gallus gallus, K locus, IGLL1, ev21

## Abstract

**Background:**

The red junglefowl, the wild outgroup of domestic chickens, has historically served as a reference for genomic studies of domestic chickens. These studies have provided insight into the etiology of traits of commercial importance. However, the use of a single reference genome does not capture diversity present among modern breeds, many of which have accumulated molecular changes due to drift and selection. While reference-based resequencing is well-suited to cataloging simple variants such as single-nucleotide changes and short insertions and deletions, it is mostly inadequate to discover more complex structural variation in the genome.

**Methods:**

We present a pangenome for the domestic chicken consisting of thirty assemblies of chickens from different breeds and research lines.

**Results:**

We demonstrate how this pangenome can be used to catalog structural variants present in modern breeds and untangle complex nested variation. We show that alignment of short reads from 100 diverse wild and domestic chickens to this pangenome reduces reference bias by 38%, which affects downstream genotyping results. This approach also allows for the accurate genotyping of a large and complex pair of structural variants at the K feathering locus using short reads, which would not be possible using a linear reference.

**Conclusions:**

We expect that this new paradigm of genomic reference will allow better pinpointing of exact mutations responsible for specific phenotypes, which will in turn be necessary for breeding chickens that meet new sustainability criteria and are resilient to quickly evolving pathogen threats.

**Supplementary Information:**

The online version contains supplementary material available at 10.1186/s12915-023-01758-0.

## Background

Accurately detecting sequence variation associated with traits of economic importance in the domestic chicken is a major goal of genetic research into this globally widespread dietary protein source [1]. Many groups are now genotyping chicken genomes to discover the underlying molecular basis of specific traits [2–6], but current methods, both sequence- and array-based, have unquantified limitations in assessing the underlying variation that connects many loci to studied traits. Investigations in other species into the variant sets compiled by techniques relying on existing linear references have revealed large gaps in variation discovery ability [7–10]. For the domestic chicken, improved completeness and accuracy of bioinformatic queries into this variation are of vital importance to the field, as computational experiments are rapidly becoming the venue of choice to assess the potential of artificial selection to improve qualities such as growth, nutrient digestibility, reproduction, and perhaps most importantly, immune resilience.

Current frequently employed methods for genotyping whole genomes mostly share the core strategy of aligning short reads to a reference genome derived from a single individual [11]; these references are usually compressed haploid representations of diploid genomes, with toggling of haplotypes due to haploid compression, or chimeric haploblocks due to allele mixing [12, 13]. Although these methods, given a reference genome of sufficient quality and reads of sufficient coverage, are able to capture most single-nucleotide variants (SNVs) and small insertions and deletions (indels) in populations, they can lead to reference bias [14, 15], and they consistently underestimate all types of structural variants (SVs) [8]. Furthermore, for best performance, the most accurate genotyping software [16] requires preexisting high-quality data about the distribution of polymorphic sites throughout the genome for statistical calibration [17] or model training [18], information that does not exist for most species. Large-scale long-read resequencing can mitigate some of these limitations [19], but the high cost and low accuracy of long reads compared to short reads, and the large amount of existing publicly available short-read sequencing data — for chicken, there are over 40,000 short-read experiments on the SRA at the time of writing but fewer than 500 long read experiments — make a full transition to the use of long reads for resequencing studies unlikely in the near future. Although there have been improvements in algorithms for using inexpensive data such as short reads for SV detection, these methods have high false positive and false negative rates [7], so previous studies of SVs in chicken using these methods [4, 20] are likely both incomplete and inaccurate.

To counter these limitations, several methods have been developed to create and use pangenome graphs as references [21–25]. A pangenome graph is a data structure that encodes the sequence and variation present among the genomes of multiple individuals [26]. Whereas a linear reference usually contains only the compressed sequence of a single individual, a pangenome includes sequence common to all individuals as well as information about the position, alleles, and frequencies of each variant site within the input assemblies. The recent publication of a draft pangenome for human demonstrated that this new paradigm allows recovery of much sequence that appears with nonnegligible frequency in the genomes of individuals across the species but is missing from even the telomere-to-telomere linear reference [27].

Alignment of short reads to a pangenome reference instead of a linear reference has been demonstrated in humans and other species, including birds, to recapitulate and improve downstream genotype calling accuracy for both small variants (i.e., SNPs and small indels) and larger structural variants [9, 28, 29]. Large insertions are nearly uncallable when using short reads aligned to linear references, with the recall of tools such as Delly [30] falling to zero for insertions larger than 400 bp, whereas graph-based tools such as VG [28] and paragraph [22] are mostly unaffected by variant length. The human pangenome’s demonstrations of improvements in read mapping, small variant genotyping, novel variant discovery, SV genotyping, and representation of complex variants [27] show the potential of this new paradigm for genome references.

In chicken, multiple alignments of reference-guided short-read assemblies [31] and de novo assemblies of high-error PacBio CLR reads [32] have revealed sequences present among chickens worldwide but missing from current references, as well as other previously unknown SVs. However, although these whole-genome alignments were both described as pangenomes by their respective authors, neither study generated a pangenome graph that can be used by other researchers as a reference for alignment to overcome the limitations presented by reference bias and difficulty in capturing SVs. They are further limited by their reliance on short reads or low-accuracy long reads, respectively, for assembly.

In this study, we generate a pangenome graph of 30 highly continuous genome assemblies of various chicken breeds, including broilers, layers, and research lines. We use this pangenome to catalog variation present in the input assemblies, including variation that was not detectable in studies using other methods, focussing on SVs in an immune system gene and a feathering-related locus as illustrations. We then go on to align short reads from 100 chickens to the graph, showing the improved performance of this method for alignment accuracy and genotyping recall compared to linear reference alignment. We expect that adoption of this new resource will allow better results in genotyping in future studies, with a goal to move toward more effective uses of chicken genome references and in the process significantly improve researchers’ ability to discover the molecular mechanisms that determine bird healthiness.

## Results

### Selection of chromosome-level assemblies

To build assembly-based pangenome references, we used the five most continuous chromosome-level assemblies of the domestic chicken currently available, along with alternate haplotypes as applicable, and new contig-level assemblies of thirteen additional chickens, most of them locally resolved into haplotypes. The five chromosome-level assemblies have contig N50 values ranging from 5.47 to 91.3 Mb (see Table 1). This includes the current species reference assembly on NCBI RefSeq, bGalGal1b, also known as GRCg7b (contig N50 = 18.8 Mb), a fully haplotype-resolved assembly of a commercial broiler line created using the trio-binning method and an F1 cross between a representative commercial broiler and a white leghorn layer [33]. bGalGal1b, as the current RefSeq reference assembly, is fully annotated, so we use it as the source of annotations in this study. Because this assembly was made using trio-binning, its creation also resulted in a fully haplotype-resolved assembly of the genetic contribution of the other parent, a white leghorn layer. We refer to this assembly as bGalGal1w, and it is also known as GRCg7w and we use both assemblies in our pangenome.

**Table 1:** The five chromosome-level assemblies used as a base for creation of pangenome references for the domestic chicken

**ID**	**Assembled bird**	**Accession**	**Ref**	**Contig N50 (Mb)**	**Contig count**
bGalGal1b	Commercial broiler	GCA_016699485.1	[33]	18.8	677
bGalGal1w	White leghorn layer	GCA_016700215.2	[33]	17.7	685
bGalGal4	Ross broiler	GCA_027557775.1	N/A	5.47	812
bGalGal5	Cobb broiler	GCA_027408225.1	N/A	8.33	712
Huxu	Huxu broiler	GCA_024206055.1	[34]	91.3	54

We sequenced and assembled to the chromosome level the genomes of two additional broilers from the Ross (Aviagen) and Cobb (Cobb-Vantress) lines, among the most commercially relevant broiler lines worldwide, to capture more of the diversity present among commercial lines of domestic chickens, and to take advantage of advances in sequencing that have occurred since the assembly of bGalGal1b and bGalGal1w, especially base-calling improvements in PacBio’s HiFi/Circular Consensus Sequence (CCS) technology. HiFi reads are accurate enough to allow the hifiasm algorithm to assemble phased contigs for two pseudohaplotypes [35], so although we only assembled the contigs from the primary assemblies into chromosomes, we used the alternate contigs during pangenome construction as well to take full advantage of their individual haploid diversity.

We also integrated the first nearly complete assembly of a chicken [34]. This assembly is of a Huxu, a Chinese broiler breed, and we refer to it as “huxu”.

Finally, we sequenced and assembled both haplotypes of 13 additional chickens to a contig level using HiFi sequencing (Additional file 1: Table 1). These chickens include research lines bred to study immune function as well as domestic breeds originating in Spain and Egypt. We produced sequencing coverage of at least 25 × (mean 35 ×) for each bird based on a genome size of 1.1 Gb. Using the hifiasm assembler, which is able to take advantage of the high accuracy of HiFi reads to create two locally phased haploid assemblies for each diploid individual sequenced, we successfully generated two haploid contig-level assemblies for each of 10 out of 13 birds. The remaining three birds are all highly inbred research lines, so their haplotypes are mostly indistinguishable and thus not able to be phased. Therefore, we used the primary assembly output of hifiasm for these. As a result, the pangenome graph includes phased haploid assemblies as well compressed diploid assemblies of these three highly inbred birds. In total, this resulted in 23 assemblies with a minimum contig N50 of 11 Mb (mean 15 Mb).

Together, these 30 assemblies represent a diverse set of domestic chickens, including commercial lines, research lines, and broiler and layer breeds originating on three continents. They also were assembled using three different techniques: haplotype-resolved trio-binning of PacBio CLR reads from the F1 offspring of a cross between two breeds (bGalGal1b and bGalGal1w), PacBio HiFi haplotype-resolved assembly (bGalGal4, bGalGal5, and additional chickens), and the current best-practice de novo assembly technique using a combination of PacBio HiFi and Oxford Nanopore Ultralong (ONT UL) reads (huxu) [34]. Although collectively these genomes do not come close to fully capturing the diversity of domestic chickens worldwide, they provide a good working template of a first pangenome reference of the domestic chicken genome.

### Creation of pangenome references

We constructed pangenome references of the chicken genome using two different methods, both used by the Human Pangenome Reference Consortium [27]: PanGenome Graph Builder (PGGB) [27] and minigraph-cactus [36]. PGGB and minigraph-cactus both take multiple assemblies as input, perform whole-genome alignments on them, and derive a pangenome graph from these alignments. However, these two pipelines differ in their fundamental approach: PGGB first performs reference-free multiple sequence alignment of all input sequences and then infers a graph using these alignments, whereas minigraph-cactus uses a single reference chosen by the user as a backbone and then progressively adds complexity to the graph by aligning the other sequences. We made a preliminary graph using each method and five chromosome-level assemblies (Table 1). For minigraph-cactus, we then created a 30-assembly graph using these five chromosome-level assemblies as well as the contig-level alternate haplotype assemblies of bGalGal4 and bGalGal5 and assemblies of both haplotypes of thirteen additional chickens from HiFi data (Additional file 1: Supplementary Table 1).

Due to the computational intractability of the PGGB graph as a reference for short-read alignment, as we discuss in subsequent subsections, we did not create a 30-assembly graph with PGGB, and used only the minigraph-cactus graph for most downstream applications. Nonetheless, we describe the PGGB graph in this section and refer to it occasionally thereafter for the sake of comparison. Therefore, the final two graphs we tested were the 5-assembly PGGB graph and the 30-assembly minigraph-cactus graph. We used the minigraph-cactus graph for most downstream analyses.

The minigraph-cactus pangenome graph contains 49 million nodes and 67 million edges, and therefore a mean degree, or the number of edges attached to a node, of 1.4. The total length of sequence represented in the graph — that is, the sum of the lengths of all nodes in the graph — is 1.13 Gb. The combined length of nodes traversed by the most complete assembly, Huxu, is 1.02 Gb. This is smaller than the 1.10 Gb total size of the assembly. This difference is because a path can traverse the same sequence in the graph multiple times. For example, in the case of a very simple graph containing three nodes, A, B, and C, a haplotype containing a duplication of B would have a path length of (A + 2B + C), whereas the total amount of sequence in the graph would be only (A + B + C). Therefore, there is in total 0.11 Gb (9.9%) of additional sequence in the graph compared to the total length of the nodes traversed by the most complete assembly. Of the other assemblies, bGalGal1b contributes the most additional sequence, 55.6 Mb, to the graph, whereas some assemblies contribute as little as 200 kb of additional sequence as a result of their relatedness to others (Additional file 2: Supplementary Fig. S1).

The PGGB pangenome graph contains 33 million nodes and 45 million edges, and therefore also a mean degree of 1.4. We found that parameter choice had a large effect on the numbers of nodes and edges, as well as the maximum degree, although not the mean degree (Additional file 2: Supplementary Fig. S2). By contrast, we used only default parameters for minigraph-cactus other than those pertaining to input and output.

Although the PGGB pangenome was made up of only five assemblies instead of 30, it contains more sequence than the minigraph-cactus pangenome: the total length of sequence represented in the PGGB graph is 1.23 Gb, compared to 1.13 Gb for the minigraph-cactus graph. This represents an additional 147 Mb or 12.0% of sequence compared to the total length of graph nodes in the Huxu genome (1.09 Gb). The 109 Mb of additional sequence is closer to previous estimates of total variation in diverse groups of chickens [37–40] than 147 Mb, suggesting possible overestimation by PGGB. The structures of these respective graphs are visibly different at the chromosome level in some places, such as at the beginning of chr13 (Additional file 2: Supplementary Fig. S3).

With the exception of the two sex chromosomes, only one of which can be present in each haplotype, all haplotypes are represented in all of the chromosome communities or subgraphs of both graphs; however, the presence of gaps in all assemblies except for Huxu means that there are places in all chromosomes where one or more haplotype paths is missing. In the PGGB graph, none of the contigs unassigned to chromosomes were included in the communities used to make the initial alignments, and thus all unassigned sequences were excluded from the pangenome graph. In contrast, in the minigraph-cactus graph, all sequences from all assemblies were included in the initial alignments. For all assemblies except Huxu, for which there is no unassigned sequence, a mean of 20.9 unassigned contigs containing a per-assembly total of 1.17 Mb of sequence were not aligned to chromosome subgraphs in the final graph.

### Cataloging of variants present in input assemblies

**Fig. 1:**
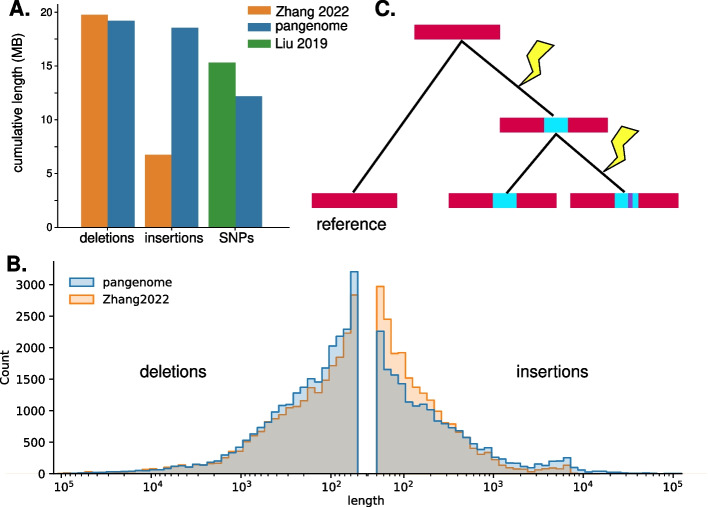
Cataloging variation in the pangenome graph. **A** Total lengths of sequence contained in insertions (INS), deletions (DEL), and SNVs, compared between this study (“pangenome”) and read-alignment methods [38, 39]. **B** Distribution of lengths of insertions and deletions found in this study compared to those found by Zhang et al. [38] using long reads shows that although long-read alignment finds more short insertions (< 1 kb) than the pangenome, the larger cumulative length of insertions found by our pangenome compared to Zhang as shown in **A** is driven by long insertions (> 1 kb), which have a larger effect on cumulative length. **C** A hypothetical schematic of how nested variation can evolve: an insertion mutation is followed by a later single-nucleotide mutation, resulting in an insertion relative to the reference that contains a segregating site. A genotype against a linear reference would represent these as three different alleles, whereas a pangenome conserves the nested structure of this variation

A pangenome graph contains the variation present in the input assemblies and can thus be used to genotype the input assemblies compared to one chosen as a reference, based on deviations from this reference path. We chose bGalGal1b for the reference as it is the highest-quality RefSeq-annotated chicken reference genome currently available. In total, we found 15 million variants in the minigraph-cactus graph present in at least one of the other 29 haplotypes compared to bGalGal1b. Twelve million of these variants are SNVs (Fig. 1a). This is a smaller number of total SNVs than has been detected in large panel studies [39, 40], which is likely a result of the smaller sample size of our experiment, with 30 haplotypes compared to 678 in [39]. We found a similar total length of deleted sequence, 19.2 Mb, as a previous study based on long read alignments, 19.7 Mb [38]. However, we were able to recover 18.5 Mb of inserted sequence, whereas the previous study recovered only 6.74 Mb [38] (Fig. 1a). Although distributions of lengths of deletions found previously by read alignment and by our pangenome method were broadly similar, we found more long insertions than was possible with long-read alignment (Fig. 1b).

**Fig. 2:**
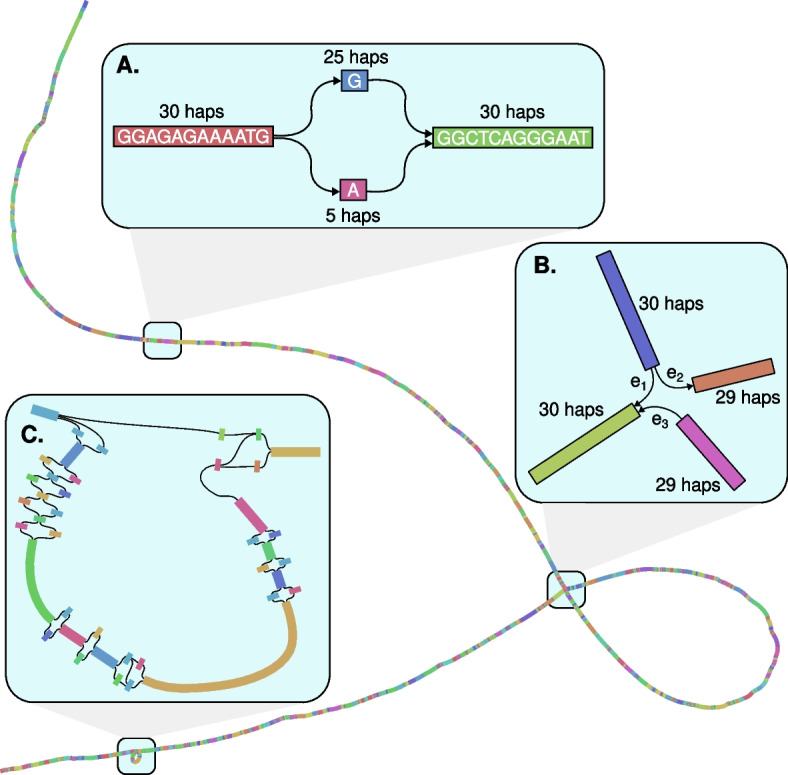
A visual representation of the pangenome graph for the gene *IGLL1*. **A ***IGLL1* contains many SNVs, including one at bGalGal1b#chr15:7,955,357, in its coding sequence. The graph of this SNV shows that although all 30 haplotypes have the same sequence before and after the SNV, 25 haplotypes have G in this position and 5 have A. **B** The pangenome of *IGLL1* contains a ~5 kb deletion compared to bGalGal1b in one haplotype of a single individual, UCD312. At the juncture in the pangenome graph where the deletion haplotype branches from the rest, this haplotype follows edge e1 to skip the sequence in the loop, whereas the other 29 haplotypes follow edge e2 to include the sequence, and then e3 to join back with the deletion haplotype afterwards. **C ***IGLL1* also contains a ~300 bp insertion compared to bGalGal1b in 22 haplotypes. The inserted sequence contains SNVs, so while a linear representation of this insertion considers each version of the insertion as a different allele, the pangenome graph is able to correctly record it as a biallelic variant (i.e., insertion or no insertion) containing additional variable sites. Furthermore, reads can align to this sequence in the pangenome but would be left unmapped when aligning to bGalGal1b as it does not contain this sequence

The B cell receptor gene *IGLL1*, which has been used as a marker for plasma B cells in chicken [41], contains examples of these different kinds of variation. The overall structure of the pangenome graph of *IGLL1* shows that there are many small variants (SNVs and indels < 50 bp), as well as two SVs longer than 50 bp (Fig. 2). By encoding the presence of small variants and their allele frequencies into the reference (Fig. 2a), alignment to pangenomes has been shown to reduce reference bias compared to a linear reference [21], which we confirm below for our chicken pangenome. For example, for the SNV shown in Fig. 2a, short reads containing the non-reference allele are in less danger of mapping incorrectly as the aligner is aware of the 17% (5/30) chance of an A in this position of the genome.

The larger of the two SVs in the pangenome graph of *IGLL1* is a ~ 5 kb deletion relative to bGalGal1b present in only one haplotype of one chicken, UCD312 (Fig. 2b). By recording this low-frequency deletion in the reference, the pangenome method ensures that reads from resequenced chickens containing the deletion are able to map to both flanking sequences through edge e1 without splitting, which would introduce a potential source of error.

Finally, a ~ 300 bp insertion relative to bGalGal1b demonstrates how a pangenome graph is able to losslessly represent nested variation (Fig. 2c). The SNVs and indels within the inserted sequence are encoded in the exact same way as they would be in reference sequence, giving a full picture of the variation present in this region.

### Disentangling a tandem repeat and viral insertion at the K locus

The K locus, short for “short wing” (*kürzer Flügel*), is a region of chrZ with an early feathering (EF) allele and a late feathering (LF) allele [42, 43]. The EF allele contains single copies of the genes *PRLR* and *SPEF2*. The LF allele contains a tandem duplication of parts of both genes [44], and often, but not always [45, 46], an insertion of the sequence of the avian leukosis virus ev21. The reference genome bGalGal1b has the EF allele and no ev21 insertion, so genotyping the K locus in other chickens using this reference is difficult because ev21 has a length of over 7kbp [46], an order of magnitude longer than the maximum insertion size that can be genotyped with short reads and a linear reference [28]. As such, it is a region that can be more accurately genotyped with the use of a pangenome graph approach.

**Fig. 3:**
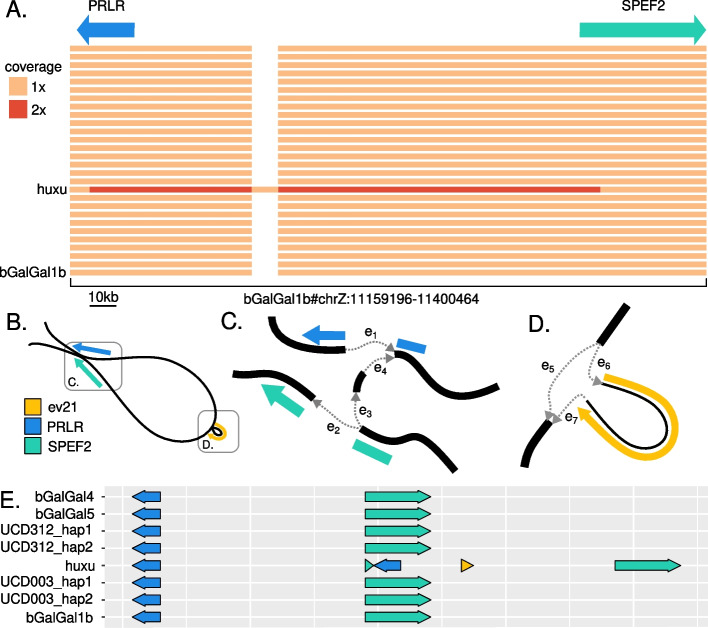
Disentangling complex variation at the K locus with the pangenome graph. **A** A one-dimensional view of the pangenome subgraph for the K locus, with nodes colored by path coverage (i.e., the number of times a haplotype path passes through them) and the locations of the genes *PRLR* and *SPEF2* denoted. Huxu shows double path coverage of part of the locus, as well as an insertion. Alignment verified that this insertion contains the sequence of the avian leukosis virus ev21. **B** A two-dimensional view of the same graph, showing both the tandem duplication and the ev21 insertion. **C** At the junction where the paths containing the tandem duplication deviate from the paths that do not, all paths begin by traversing edge e1 and moving through most of the sequence of the K locus. However, at the e2/e3 fork, a path can either traverse e2 to leave the K locus, or traverse e3 and e4 to include a tandem duplication of parts of *PRLR* and *SPEF2*. **D** A more detailed view of the ev21 insertion, showing the two possible paths at this juncture: a path can traverse edge e5 to skip the insertion, or it can traverse edge e6, then the ev21 sequence, then e7, to include the insertion. **E** Linear untangled view of the locus, confirming previous studies of the structure of the locus, with a tandem duplication of parts of both genes and an insertion of the ev21 sequence

We first created a one-dimensional representation of the minigraph-cactus pangenome graph structure of the K locus colored by path coverage, as a node through which the same haplotype path travels more than once indicates a duplication (Fig. 3a). This representation shows that although most of the haplotypes represented in the pangenome graph contain only one copy of this locus, Huxu has a duplicated region and an insertion. The 2× path coverage region in Huxu covers parts of both *PRLR* and *SPEF2*, consistent with the tandem duplication found by Elferink et al. [44]. We also found a misassembly in bGalGal1w, with unassigned scaffolds containing the sequence (see Additional file 2: Supplementary Note 1 [33, 44] and Supplementary Fig. S4). Furthermore, Huxu contains an insertion relative to the reference sequence bGalGal1b. Alignment verified that the inserted sequence is the ev21 viral genome.

Next, to better understand the structure of the locus, we created a two-dimensional representation of the graph at this locus (Fig. 3b–d). This representation of the graph shows the tandem duplication as a junction where a path can either leave the K locus or repeat it (Fig. 3c), and the insertion as a loop containing the ev21 genome covered only by Huxu (Fig. 3d).

Finally, to view the alleles linearly, we used the “untangle” function of ODGI [24] to lay out each haplotype of the minigraph-cactus graph (Fig. 3e). The resulting gene layout of the two alleles is consistent with previous knowledge about the structure of the locus [44–46].

### Genotyping ALVEs in the pangenome graph

In addition to the ev21 insertion present in some alleles of the K locus, chickens carry other endogenous retroviral insertions of avian leukosis virus subgroup E (ALVE) [47]. Many of these viral insertions remain at least partially functional, retaining their ability to express individual viral proteins or even create full viral particles [48]. The presence of some of these insertions in the chicken genome has been shown to be associated with phenotypic traits such as egg production [49], plumage color [50], and disease susceptibility [51]. As such, these insertions represent structural variants with known phenotypic effects, so we searched for and genotyped them in our pangenome graph.

Including ev21, we found 18 ALVEs common in commercial layers and broilers (Additional file 2: Supplementary Fig. S5). Most (12/18) of these ALVEs are present in only one haplotype, but others are present in up to 20 haplotypes (ALVE1). ALVE-TYR, present in 3 of the 30 haplotypes in the pangenome, disrupts the *Tyrosinase* gene, causing a recessive white phenotype and reductions in growth rate of muscle mass [52]. Two of the genes in ALVE3, *gag* and *env*, present in seven haplotypes, are known to be highly expressed due to their placement within an intron of the non-viral *HCK* gene. This expression offers some degree of protection from exogenous avian leukosis virus infection through receptor interference [53], but can also lead to immune tolerance, with lower antibody production and higher mortality [54].

### Use as a reference for resequencing and genotyping

Given the improvements in accuracy and recall of genotyping shown in other species by using pangenome graph-based methods, we set out to demonstrate the usefulness of our pangenome representations for alignment and genotyping. For this, we used simulated short reads as well as short reads from 100 domestic and wild chickens (Additional file 3: Supplementary Table 2). For comparison between linear and graph-based methods, we called genotypes using both linear alignments to bGalGal1b as well as graph alignments to our pangenomes.

**Fig. 4:**
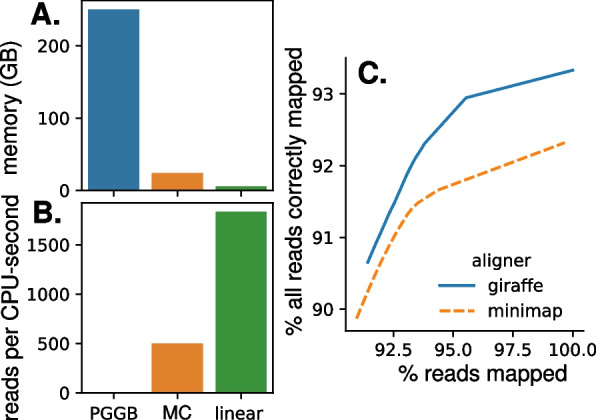
Comparing pangenome and linear aligner performance for short reads. **A**, **B** Alignment of short reads with VG giraffe is more memory-efficient (**A**) and faster (**B**) when aligning to the minigraph-cactus (MC) pangenome graph compared to the PGGB graph. Linear alignment with minimap2 is the fastest and most memory-efficient. **C** A larger percentage of all simulated reads is correctly aligned with giraffe regardless of how permissive the minimum map quality filter is

For downstream use by existing short-read genotype callers, alignments must be converted from graph coordinates to linear coordinates; this process is called surjection. Alignment of short reads to the PGGB graph and surjection to bGalGal1b was infeasible, with a throughput of only 1.6 reads per CPU-second on a test set of 10 k paired-end reads, and inability to complete alignment of a larger test set of 1 M paired-end reads without running out of memory with 250 GB allocated to the job. Further investigation revealed that surjection was the bottleneck, as graph alignment without subsequent surjection had a throughput of 147 reads per CPU-second and a maximum memory usage of 31 GB for the 1 M test set. By comparison, alignment of the 1 M test set to the minigraph-cactus graph followed by surjection to bGalGal1b had a throughput of 500 reads per CPU-second and a maximum memory usage of 24 GB, and minimap2 could align 1832 reads per CPU-second to bGalGal1b with 5.4 GB memory (Fig. 4a, b).

To compare accuracy of graph alignment to linear alignment, we simulated one million pairs of paired-end reads through sampling from the graph with random errors added, and aligned them to both the cactus-minigraph pangenome with VG giraffe [9] and the linear bGalGal1b reference with minimap2. We then determined the accuracy of the alignments by comparing the location to which reads were aligned to the location from which they were sampled. Giraffe performed better than minimap at every level of stringency, based on what percentage of all reads were mapped correctly (Fig. 4c).

**Fig. 5:**
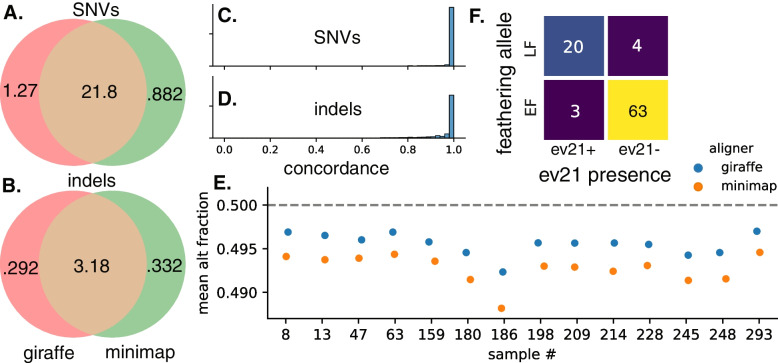
Genotyping 100 diverse chickens. **A**, **B** Counts in millions of common and different SNVs (**A**) and indels (**B**) found by genotyping pipelines using giraffe vs. minimap as the aligner. Only variants with a quality score of at least 10 are considered. **C**, **D** Concordance distributions for SNVs (**C**) and indels (**D**) detected by both genotyping methods with QUAL ≥ 10. **E** Mean fractions per sample of mapped reads containing the alternate allele at putative heterozygous sites show that giraffe alignments contain less reference bias for every chicken, as they deviate less from the expected value of 0.5. Sample information in Additional file 3: Supplementary Table 2 and full plot for all 100 chickens in Additional file 2: Supplementary Fig. S6. **F** Genotyping 100 chickens at the K locus reproduces previous results finding that although most chickens with the late feathering allele (LF) also have an ev21 insertion at the K locus (ev21+), some chickens have the late feathering allele without an ev21 insertion

To test the downstream effects of these differences in mapping accuracy, we genotyped 100 chickens from diverse breeds using both giraffe pangenome alignments and minimap linear alignments of 10–15× coverage short reads, and compared the results between the two methods (Fig. 5). Whereas the two methods found similar sets of SNVs (Fig. 5a) and indels (Fig. 5b), there were substantial differences. Agreement was unsurprisingly higher for SNVs, although the pipeline using giraffe alignments found a larger number with a quality score of at least 10 than the pipeline using minimap (Fig. 5a). For variants found by both methods, per-sample SNV concordance had a mean of 97.9% with a standard deviation of 9.1% (Fig. 5c). Indel concordance was lower, with a mean of 94.0% and a standard deviation of 12.9% (Fig. 5d).

To determine whether reference bias is a factor in the different genotyping results between the two methods, we examined the proportion of mapped reads containing the reference allele at putative heterozygous SNV sites. Reference bias across these sites, which we define as the difference between the mean fraction of reads containing the alternate allele and the expected alternate allele fraction of 0.5, is lower for all of the 100 chickens when using pangenome alignment instead of linear alignment, with a mean reference bias reduction of 38% (Fig. 5e, Additional file 2: Supplementary Fig. S6).

To connect the genotypes of these chickens to the geographic origins of their breeds, we performed principal component analysis (PCA). Although there is not complete separation of geographic origins on the PCA plot, as expected due to admixture, American and Northern European breeds fall into narrow bands on PCs 1 and 3, respectively, while Asian breeds are more diverse (Additional file 2: Supplemental Fig. S7).

Finally, we used the short-read alignments to the pangenome graph to genotype the K locus based on edge coverage (Fig. 5f). All of these chickens are female and thus only have one copy of the Z-linked K locus. Of the 100 chickens, 23 have the ev21 insertion (ev21+) and 24 have the tandem repeat (late feathering/LF). As found in previous studies [45, 46], the ev21 insertion and the tandem duplication are not inextricably linked, although they do usually appear together: three chickens, all standard Rhode Island breeds, have the ev21 insertion but not the tandem repeat, and four chickens, two Silkies and two Cochins, have the tandem repeat but not the ev21 insertion.

## Discussion

With the quickly accumulating numbers of haplotype-resolved genomes for many species, the pangenome model of integrated presentation of within-species variation stands to become ubiquitous [26, 27]. Such resources already exist for other livestock such as swine [55] and cattle [56, 57]. One of the greatest advantages of pangenome references in other species has been the capture of sequences not present in linear reference genomes. Compared to the nearly complete assembly of the Huxu chicken genome, our pangenome graph contains 109 Mb of additional sequence. Some of this additional sequence comes from SNVs or small indels that are relatively straightforward to represent in the context of a linear reference, and some of it is made up of nodes whose sequences are similar to nodes traversed by the Huxu assembly, but are represented separately. Thus, the true accessory genome length is likely less than 109 Mb compared to Huxu. Nonetheless, the tripling of total insertion length detectable using this pangenome compared to using long-read alignments as in a previous study [38] shows that much of this additional sequence is made up of variation that cannot be represented in a traditional linear reference genome, and therefore, many reads from these regions of the genome cannot be mapped to a linear reference because it does not contain the parts of the genome the reads came from. By adding additional assembled chicken genomes of more diverse origins this amount of novel sequence will grow.

Other studies have presented multiple alignments of chickens as pangenomes [31, 32], but our graph-based approach, which uses assemblies based on long and highly accurate PacBio HiFi reads as well as one near-complete assembly, allows the pangenome to be used not just as a method for cataloging variation present in the input assemblies, but also as a reference for future resequencing studies. By comparing pipelines using linear versus pangenome alignments of short reads to genotype 100 chickens from diverse breeds, we demonstrated the improved alignment performance of pangenome alignment over linear alignment and showed the downstream effects of these improvements on genotyping. Unfortunately, there does not yet exist a high-confidence truth set of variant calls for chickens as there does for humans [16], so we cannot compare the accuracy of these differing genotype calls. Nonetheless, given the improvements in alignment performance we have shown in chicken with both simulated and real reads, and the improvements in genotyping demonstrated in human and yeast by using the giraffe pangenome aligner [9, 27], we predict that the genotypes we inferred using giraffe pangenome alignment are substantially more accurate than those we inferred using linear alignment.

We tested two approaches for creating pangenome graphs, PGGB and minigraph-cactus, which were both used by the Human Pangenome Reference Consortium to create the first draft of the human pangenome [27]. These two methods each have advantages and disadvantages which we explore here. PGGB uses a reference-free approach, whereas minigraph-cactus makes alignments to a single reference. The single-reference approach of minigraph-cactus greatly increases the efficiency of alignment to the graph, but also results in some regions of non-reference sequence being clipped, as shown in Additional file 2: Supplementary Fig. S3. Furthermore, minigraph-cactus is able to choose most alignment parameters automatically, whereas PGGB results are highly dependent on parameter choice. Due to the absence of a deterministic process for choosing best parameters or even evaluating and comparing graphs made with different parameters, the ability of minigraph-cactus to automatically choose alignment parameters presents an advantage over PGGB. In the end, our choice of the minigraph-cactus graph for most downstream analyses was primarily based on the computational intractability of the PGGB graph for use as an alignment reference; regardless of accuracy or completeness, a graph to which only 1.6 reads per CPU-second can be aligned is not usable for most purposes with the resources currently available to most genomics researchers.

Our determination of the structure of the K locus and subsequent genotyping demonstrates the power of pangenome graphs in the study of loci containing complex structural variants. The initial discovery of the insertion of an endogenous avian leukosis virus in the late feathering allele required cell culture work [58], and a later study establishing the tandem repeat [44] necessitated extensive quantitative PCR experiments targeted at 20 different segments of the locus. Although the latter was performed after a linear reference genome was available, this reference, like all subsequent versions of the reference genome for chicken, contains the early feathering allele and no ev21 insertion at the K locus, and no current method can reliably genotype SVs of this size using short reads and a linear reference [28]. More recent work on the relationship between the ev21 insertion and the late feathering phenotype, though undertaken after improved reference genomes and large amounts of public sequencing data from different breeds of chickens became available, also relied on targeted PCR [45, 46]. In contrast, we were able to replicate these findings using only existing short-read whole-genome sequencing data and pangenome methods. We expect that our pangenome, and future pangenomes using telomere-to-telomere genome assemblies, which exist for increasing numbers of species [59–63] but not yet chickens, will enable discoveries about complex structural variation at important immune loci such as the major histocompatibility complex (MHC) and T cell receptor gene (TCR), providing insight into the genetic diversity necessary to fight evolving pathogen threats in this major worldwide source of protein, which also threaten wildlife with increasing frequency [64].

The tool used by both the minigraph-cactus and PGGB pipelines to produce a VCF of the input assemblies based on the graph, vg deconstruct, does not currently classify SVs based on type, e.g., as inversions or translocations, but instead represents all SVs as either inserted or deleted sequence. Therefore, a complex variant such as a translocation is represented as a deletion of sequence in one location and an insertion of the same sequence in another location. We detected the tandem duplication present at the K locus through manual examination of graph structure. Tools such as vcfwave [65] are able to secondarily reclassify these complex structural variants properly, but due to the rapidly changing nature of software in this field, we report SVs only as insertions and deletions. We expect future versions of this pangenome to use new tools to report inversions and translocations as well.

For the most part, we were able to use best practices established by the human pangenome reference consortium [27] for the creation and use of this pangenome. However, in some cases, such as our inclusion of highly inbred research lines that could not be phased due to the similarity of their haplotypes, there is no available precedent from the human pangenome. As pangenomes are built for more species, we hope to see consensus emerge about best practices for cases such as this that do not apply to humans.

We created this first draft of the chicken pangenome out of a mixture of commercial and research lines and previously published reference assemblies. Despite this somewhat arbitrary sampling process based mostly on sample availability, using the pangenome as a reference increases accuracy, decreases reference bias, and makes it possible to genotype structural variants that are too large and complex to genotype with a linear reference and short reads. Nonetheless, we expect future versions to improve these measures even further through the inclusion of more chickens, sampled more strategically, to best capture the full diversity and variant frequencies of chickens worldwide.

## Conclusions

In this paper, we have presented the first pangenome graph reference for the domestic chicken. We show its utility as a catalog of variation, including structural variation too large or complex to be detected using previous methods, and as a reference for the alignment of short reads. Given the improvements we have demonstrated in this model over a linear reference, we expect this pangenome, and new versions with additional broadly diverse chicken breeds incorporated, to serve as a resource to the community for future resequencing studies as well as investigation of complex loci, especially in immune-related genes.

## Methods

### Sequencing and assembly of bGalGal4 and bGalGal5

One female Ross 308 (Aviagen) and one female Cobb 550 (Cobb-Vantress), both commercial broiler chickens, were euthanized in the framework of a research experiment at 38 days of age. Cardiac puncture was immediately employed to collect 12 aliquots of 100 µl of blood in tubes with EDTA and 1 ml of ethanol > 99.7% from each animal. Samples were frozen at −20 °C.

For both assemblies (bGalGal4 and bGalGal5), we followed the VGP 2.0 pipeline [12]. We generated 32× Pacbio HiFi data on a Sequel IIe, and then used cutadapt [66] to trim off adapters that were not trimmed in the Pacbio software processing. We assembled contigs using HiFiasm v0.14 [67], generating a semi-haplotyped phased primary contig and alternate contig assembly. From the primary assembly, we removed false haplotype duplication and placed them in the alternate using purge_dups v1.2.5 [68]. We then scaffolded the contigs with Bionano Genomics optical maps (319× and 459× respectively), generated on a Saphyr instrument using DLE label, with Bionano Solve. We then further scaffolded with Arima Genomics Hi-C v2 (65× and 122× respectively), using salsa v2.2 [69]. The primary assembly was then curated using gEVAL [70], structural errors corrected, and chromosomes named according to their numbers in the bGalGal1 GRC7g reference. 10X Genomics data were also generated, and used for orthogonal validation, but not scaffolding. The primary and alternate assemblies were deposited in NCBI under accession numbers GCA_027557775.1 (bGalGal4) and GCA_027408465.1 (bGalGal5), and all data are available in Genome Ark (https://genomeark.github.io/genomeark-all/Gallus_gallus/).

### Sequencing and assembly of additional chickens

High molecular weight (HMW) DNA from blood of 13 juvenile male chickens (Additional file 1: Supplementary Table 1), maintained and bled under ADOL IACUC-approved Animal Use Protocol #2019-15 for breeder management, was sequenced on the Pacific BioSciences Sequel IIe. HMW samples were sheared using a Diagenode Megarupter3 shearing device targeting 18–22 kb fragments. Libraries were prepared with the PacBio SMRTbell Prep Kit 3.0. Library size distribution was determined on the Agilent Femto Pulse and a Qubit fluorometer was used to measure concentration. Sequencing polymerase was bound to the SMRTbell libraries with the Binding Kit 3.2 and run on Sequel IIe with the Sequel II Sequencing Kit 2.0 and SMRT Cell 8 M. HiFi data was collected with Instrument Control Software Version 11.0 and Chemistry Bundle 11.0 with a movie time of 30 h. The On Plate Loading Concentration was 130pmolar.

HiFi reads for each of the chickens were assembled into contigs using hifiasm v0.18.9 [35] with default options. Both haplotypes output by hifiasm were used in subsequent analyses.

### Creation of PGGB pangenome

We constructed a pangenome reference from the five input assemblies bGalGal1b, bGalGal1w, bGalGal4, bGalGal5, and HuxuT2T (Table “assemblies”). First, we extracted chromosome sequences from the assemblies and gave them names according to the PanSN-spec, in the format of “[assembly name]#[chromosome name]”, e.g., “bGalGal4#chr5”. The PGGB pipeline recommends first partitioning the assemblies into communities, where each community is a set of sequences that should be aligned to each other, for example, all sequences from each assembly assigned to the same chromosome. We partitioned the assemblies into 41 communities, one for each chromosome based on whole-genome alignments made with mashmap [71] in one-to-one mode and a percent identity cutoff of 90%, and then constructed a pangenome graph for each chromosome separately. Due to disagreements in the naming of microchromosomes among the five assemblies, some of the communities contain chromosomes named differently in the different assemblies (Additional file 4: Supplementary Table 3).

For every chromosome, we constructed its pangenome graph using the Pangenome Graph Builder (PGGB) v0.4.1 [27]. Briefly, this pipeline uses wfmash v0.9.1 [72] to align the input assemblies, seqwish v0.7.6 [25] to build a graph from the alignments, smoothxg v0.6.5 [73] and gfaffix v0.1.3 [74] to clean up the graph, and odgi v0.7.3 [24] to visualize the graph. We first ran pggb with default parameters, except for parameter “-n” set to the number of assemblies being aligned for the chromosome in question (this number is five for most chromosomes, with the exception of sex chromosomes and some microchromosomes without full representation in all five assemblies) and “-G 3079,3559”. For postprocessing and optimal visualization, we redrew the 2D graph visualization using the odgi draw command with parameters “-C -w1000,” and we redrew the 1D graph visualization by first resorting the graph based on positions in the bGalGal5 path using the command odgi sort with parameters ‘-H < (echo “bGalGal5#${chromosome_name}”) -Y’ and then drawing with the odgi viz command with default parameters.

To find the optimal parameters for each chromosome, we performed a parameter sweep of the segment length (-s), mapping percent identity (-p), and minimum match length (-k) options to the pggb command. We tested every member of the cartesian product set of the parameter values s = {5 k, 10 k, 30 k, 50 k, 80 k}, p = {85, 90, 94,97}, and k = {10, 19, 50, 100, 150}. We evaluated the results as suggested in PGGB documentation, using a combination of examination of graph statistics, especially node count and maximum degree, with the odgi stats command and visual inspection of the graph structure using the odgi viz output. For some microchromosomes, we made more granular adjustments to the parameters to fine-tune their graphs. Additional file 4: Supplementary Table 3 shows the final parameters chosen for each chromosome.

Finally, we created a single pangenome graph containing the respective connected component for each community using the odgi squeeze command with default parameters. This resulted in a single graph file with extension “.og” that is easily convertible to other sequence graph formats such as GFA and VG.

### Creation of minigraph-cactus pangenome

We ran the minigraph-cactus pipeline [36] using the cactus v2.4.2 Docker image and a nextflow pipeline built for this purpose [75]. As input, we used the five chromosome-level assemblies in Table 1, the alternate haplotypes of bGalGal4 and bGalGal5, and both haplotype assemblies of an additional 13 chickens listed in Additional file 1: Supplementary Table 1. We specified bGalGal1b as the reference, because although it is not the highest-quality assembly, it is the best RefSeq-annotated assembly on NCBI, so we wanted to call variants against it downstream.

### Additional sequence analysis

We determined the amount of additional sequence contributed to the graph by each sample through an iterative process. First, we removed all nodes traversed by the Huxu assembly from the graph as it is the most complete assembly. Then, for each remaining bird, we summed up the length of all nodes traversed by either haplotype of this bird, found the bird with the largest sum, and removed all nodes traversed by this bird’s haplotypes from the graph. We repeated this process until there were no samples remaining. The python program we wrote for this purpose is included in the repository cited in the Code Availability statement.

### Format conversions and subgraph extraction

- To convert GFAv1.1 format as output by minigraph-cactus to OG format for downstream use in ODGI visualization tools, we used the command “vg convert -gfW” to convert to GFAv1.0, and then “odgi build -g -Os” to build an OG graph out of the GFAv1.0 file.
- To convert GBZ format to HG format, we used the command “vg convert”.
- To convert HG format to GFA format, we used the command “vg convert -f”.
- To convert OG format to GFA format, we used the command “odgi view -a -g”.
- To extract regions from graphs in HG format, we used the command “vg find -p ‘bGalGal1b#[chromosome]:[start]-[end]’”.
- To extract regions from graphs in OG format, we used the command “odgi extract -d0 -E -r ‘bGalGal1b#[chromosome]:[start]-[end]’”.

### Genotyping input assemblies

Both assembly-based graph construction pipelines, pggb and minigraph-cactus, can output vcf files containing genotypes for the input assemblies relative to the reference, in our case bGalGal1b. Minigraph-cactus does this by default; pggb does with the addition of the option “-V ‘bGalGal1b:#:’”. Where necessary, we concatenated vcf files for each chromosome into a single genome-wide vcf using the bcftools concat command v1.15.1 [76].

### Graph visualization

To visualize specific regions of the pangenome graph, we first looked up coordinates relative to bGalGal1b on RefSeq, extracted them from the graph, output in GFA format, and visualized using bandage v0.8.1 [77]. Commands for extraction and conversion are given under the heading “Format conversions and subgraph extraction”.

### Genotyping ALVEs

As previously described in [4th chicken report], we identified assembled Avian Leukosis Virus subgroup E (ALVE) integrations by performing a search for ALVE1 (GenBank: AY013303.1) with BLAST v2.10.0 [78] in each of the contributing fully assembled reference sequences using ALVE1 (GenBank: AY013303.1). We used flanking sequence to annotate ALVEs with known integration sites [47]. We then translated all coordinates to bGalGal1b coordinates using odgi position and looked up these insertions or deletions relative to bGalGal1b in the minigraph-cactus vcf output.

### Read simulation

We simulated reads using the “vg sim” command with a nucleotide substitution error rate of 0.24% as estimated by Pfeiffer et al. [79] and an indel error rate of 0.029% as in [9]. This command randomly samples reads from the pangenome graph and adds errors based on the specified error rates, keeping information about the location from which the reads were sampled in the read header so that it can be used to test accuracy downstream.

### Sequencing of short read chickens

We sampled 236 chickens from 62 breeding farms that specialize in heritage and rare chicken breeds in May and December 2021. In short, we collected 0.5–2 mL of blood from each bird by puncturing the brachial vein with a syringe (gauge size 18.5–28 depending on the size of the bird). The blood was immediately expelled through the syringe into K2EDTA vacutainers and stored on dry ice. Upon arrival at the lab, the blood samples were transferred to a −80 °C freezer. DNA was extracted using the QIAamp Fast DNA Tissue Kit. Library preparation and sequencing were performed at BGI Group. Libraries were prepared using a DNA short-insert protocol for 150 bp paired-end reads and sequenced on the DNBseq platform. Seven samples failed to be sequenced due to low quality, so were excluded from further analyses. We chose a subset of 100 of these samples for the final analysis, selecting breeds that were previously genotyped at the K locus [45, 46] where available and choosing the rest by balancing the conflicting goals of including multiple chickens from the chosen breeds and having many breeds represented.

### Short read alignment

To align short reads to the PGGB graph, we first converted the graph to GFA format using the command “odgi view -g” and then converted the GFA format to GBZ format [80] and created giraffe indices from the output with the command “vg autoindex -w giraffe.” The minigraph-cactus pipeline outputs all indices necessary to run giraffe by default, so no further processing was necessary to prepare it for alignment of reads with giraffe.

To test timing and memory usage, we arbitrarily chose a publicly available set of short reads from a chicken (SRR9967588) and subsetted the first 1 million pairs. This test failed for alignment to the PGGB graph due to running out of memory, but a smaller subset of 10,000 read pairs was successful. We aligned the test set of reads to the graph using the command “vg giraffe” with arguments “-o BAM.” Because the PGGB graph does not contain a reference sequence like the minigraph-cactus graph, we additionally specified the reference chromosomes with the arguments “--ref-paths bGalGal1b_paths.tsv,” where bGalGal1b_paths.tsv is a tab-separated file containing a list of all chromosomes in bGalGal1b and their sizes. For comparison to alignment to a linear reference with minimap2 v2.24 [81], we created a short-read minimap index of bGalGal1b with the command “minimap2 -x sr -d” and then aligned reads to it with the command “minimap2 -a” piped to “samtools view -bh” with samtools v1.16.1 [76] to convert to bam format for a fair comparison, since we ran giraffe with bam output.

For alignment of short reads from 100 chickens, we ran vg giraffe with default options, outputting the results in GAM format. We surjected the GAM files to BAM format with bGalGal1b as the reference genome using the command “vg surject” with default options.

### Comparison of linear and graph alignments with simulated reads

To compare the accuracy of alignments of simulated reads between linear and graph aligners, we aligned the simulated reads both to the bGalGal1b linear reference using minimap2 and to the pangenome graph reference using giraffe, as described above. We converted the minimap2 output to GAM format using the command “vg inject,” and then compared the minimap2 and giraffe GAMs to the truth set using “vg gamcompare,” all as in [9].

### Genotyping

We genotyped the 100 chickens based on these alignments using elprep [82] v5.1.2, a multithreaded reimplementation of GATK. Briefly, we generated an elfasta sequence reference (an indexed binary form of the reference fasta for downstream use) for bGalGal1b using the command “elprep fasta-to-elfasta,” created a list of sites from the minigraph-cactus vcf output with SVs larger than 1000 bp filtered out using the command “elprep vcf-to-elsites,” and ran the “sfm” command with settings as recommended in the manual to generate a gvcf for each bird, which we then combined into a single gvcf with GATK CombineGVCFs and joint genotyped with GATK GenotypeGVCFs [17]. The location of our scripts for genotyping, as well as all other analyses in this paper, is given in the “Availability of data and materials” section.

### Genotyping method comparison

To compare the respective outputs of the giraffe- and minimap-based genotyping pipelines, we used bcftools v1.17 [76] command “isec -c some” to create four vcf files: variants only detected by the giraffe pipeline, variants only detected by the minimap pipeline, giraffe pipeline calls of variants detected by both pipelines, and minimap pipeline calls of variants detected by both pipelines. We counted variants with QUAL ≥ 10 in all of these files, subsetting by variant type with “bcftools view -v [snp|indel].” To compare the per-sample calls made by the respective methods for variants detected by both, we used “bcftools merge --force-samples” to create a single vcf containing calls made by both methods, and then used a custom python script (included in code availability) to calculate the percent agreement for each variant.

### Reference bias estimation

We estimated the amount of reference bias by calculating the mean fraction of reads mapping to putative heterozygous sites containing the alternate allele, and comparing to the expected value of 0.5. We define putative heterozygous sites as positions with coverage of at least 10× where the portion of reads containing the minor allele is at least 25%, as in [15]. Briefly, we filtered low-quality mappings and multimapping reads with “samtools view -F2304 -q10,” created pileups with “samtools mpileup -d100 –no-BAQ,” and piped the results to a custom C program to find putative heterozygous sites and calculate alternate allele frequencies at these sites. All code used to perform this analysis is in the project’s code repository.

### Principal components analysis

To visualize the shared genetic ancestry across chicken breeds, we performed a PCA using Plink 2.0 [83]. We filtered for linkage disequilibrium using the parameters “*indep 50 5 0.5*” following Dementieva et al. [84]. We grouped the samples by the geographic origin of the breed.

### K locus genotyping

To genotype the K locus, we converted each GAM file to GAF format using the command “vg convert -G” and counted reads covering the edges e1 through e7 as shown in Figure “K locus.” We used binomial tests with *p*-value cutoffs of 0.05 to assign genotypes to each chicken for both the ev21 insertion and the tandem duplication; chickens with both *p*(insertion) > 0.05 and *p*(no insertion) > 0.05 were marked as inconclusive.

### Supplementary Information

**Additional file 1: Supplementary Table 1.** Additional chickens sequenced with HiFi reads and assembled for inclusion in the minigraph-cactus pangenome.

**Additional file 2: Figure S1.** Additional sequence per sample. **Figure S2.** Effects of parameters on PGGB graph. **Figure S3.** Comparison of chr13 between PGGB and minigraph-cactus. **Figure S4.** Unplaced contigs at K locus. **Figure S5.** Genotyping ALVEs in the pangenome. **Figure S6.** Reference bias in giraffe vs. minimap alignments. **Figure S7.** PCA of short read chickens.

**Additional file 3: Supplementary Table 2.** Chickens sequenced with short reads and genotyped using pangenome graph alignments.

**Additional file 4: Supplementary Table 3.** Final parameters for PGGB for each chromosome.

## Data Availability

The datasets generated and/or analyzed in the current study are available in NCBI repositories under BioProject accessions PRJNA838369 [85], PRJNA838370 [86], PRJNA971225 [87], and PRJNA1031205 [88]. The pangenome graph, a vcf of variants present in the graph, and vcfs of the resequenced chickens genotyped using both linear and pangenome methods are available in a Zenodo repository at https://doi.org/10.5281/zenodo.10018222 [89]. The code used to perform the analysis in the current study is available on GitHub at https://github.com/WarrenLab/chicken-pangenome-paper [90].
