# Supplementary material for: A pangenome graph reference of 30 chicken genomes allows genotyping of large and complex structural variants
Source: BMC Biol. 2023 Nov 22;21:267. doi: 10.1186/s12915-023-01758-0 (PMC10664547; doi:10.1186/s12915-023-01758-0)

Supplementary Material for "A pangenome graph reference of 30 chicken genomes allows genotyping of large and complex structural variants."

Edward S. Rice[1,2], Antton Alberdi[3], James Alfieri[4], Giridhar Athrey[5], Jennifer R. Balacco[6], Philippe Bardou[7], Heath Blackmon[8], Mathieu Charles[9], Hans H. Cheng[10], Olivier Fedrigo[6], Steven R. Fiddaman[11], Giulio Formenti[6], Laurent Frantz[2,12], M. Thomas P. Gilbert[3], Cari J. Hearn[10], Erich D. Jarvis[6,13], Christophe Klopp[14], Sofia Marcos[3,16], Andrew S. Mason[15], Deborah Velez-Irizarry[10], Luohao Xu[17], Wesley C. Warren[18]*

[1] Bond Life Sciences Center, University of Missouri, Columbia, MO, USA
[2] Faculty of Veterinary Medicine, Ludwig-Maximilians-Universität, München, Germany
[3] Center for Evolutionary Hologenomics, Globe Institute, University of Copenhagen (UCPH), Copenhagen, Denmark
[4] Department of Ecology & Evolutionary Biology, Texas A&M University, College Station, TX, USA
[5] Department of Poultry Science, Texas A&M University, College Station, TX, USA
[6] Vertebrate Genome Laboratory, The Rockefeller University, New York, NY, USA
[7] Sigenae, GenPhySE, Université de Toulouse, INRAE, ENVT, F-31326, Castanet Tolosan, France
[8] Department of Biology, Texas A&M University, College Station, TX, USA
[9] University Paris-Saclay, INRAE, AgroParisTech, GABI, Sigenae, Jouy-en-Josas, France
[10] USDA, ARS, USNPRC, Avian Disease and Oncology Laboratory, East Lansing, MI, USA
[11] Department of Biology, University of Oxford, OX1 3SZ, UK
[12] School of Biological and Behavioural Sciences, Queen Mary University of London, London E1 4DQ, UK
[13] The Howard Hughes Medical Institute, Chevy Chase, MD, USA
[14] Sigenae, Genotoul Bioinfo, MIAT UR875, INRAE, Castanet Tolosan, France
[15] Department of Biology, The University of York, York, UK
[16] Applied Genomics and Bioinformatics, University of the Basque Country (UPV/EHU), Leioa, Bilbao, Spain
[17] Key Laboratory of Freshwater Fish Reproduction and Development (Ministry of Education), Key Laboratory of Aquatic Science of Chongqing, School of Life Sciences, Southwest University, Chongqing 400715, China
[18] Department of Animal Sciences, University of Missouri, Columbia, MO, USA

* To whom correspondence should be addressed: warrenwc@missouri.edu

Contents:

Supplementary Note 1
Supplementary Figures S1-S7

Supplementary Note 1

The reference genomes bGalGal1b and bGalGal1w are haploid assemblies of the two haplotypes of a female (WZ) F1 hybrid of a female (WZ) broiler and a male (ZZ) white leghorn layer, separated using trio binning. Thus, the assembled individual has a W chromosome inherited from the broiler and a Z chromosome inherited from the white leghorn layer. W-linked sequence was therefore binned with the maternal haplotype and Z-linked sequence with the paternal haplotype. However, after assembly, the assembled Z chromosome was removed from the paternal layer assembly bGalGal1w and placed in the maternal broiler assembly bGalGal1b so that bGalGal1b, intended to be the primary reference assembly for the species, could include both a W and a Z chromosome for alignment [33]. Any contigs belonging on the Z chromosome but not properly assigned to it were thus left behind in the assembly bGalGal1w, separated from the rest of the Z chromosome sequence.

Two unassigned contigs in bGalGal1w, JAENSL010000411 and JAENSL010000412, are included in the pangenome alignments of the K locus (Supplementary Figure S4a). These contigs share a 31kb overlap with 97% identity. Based on the long, high-identity overlap between the contigs, we believe they should have been joined into a single contig, but were not due to the high error rate in the PacBio CLR reads used in this assembly and the duplicated nature of this sequence making unique alignment of reads difficult during the polishing step. The new contig formed by joining these two contigs at the overlap aligns closely to the region of chrZ duplicated in Huxu, contains the ev21 insertion, and is 177.8kb long, close to the 176kb estimate of the length of the tandem duplication provided by Elferink et al. [44] (Supplementary Figure S4b). Thus, this sequence appears to be the tandem duplication present in the late feathering locus, and should have been assigned to the single Z chromosome that is part of the bGalGal1w haplotype but was included in the bGalGal1b reference for convenience (Supplementary Figure S4c).

Supplementary Figure S1. Sequence added to the graph by each sample, with samples ordered by how much sequence they contribute.

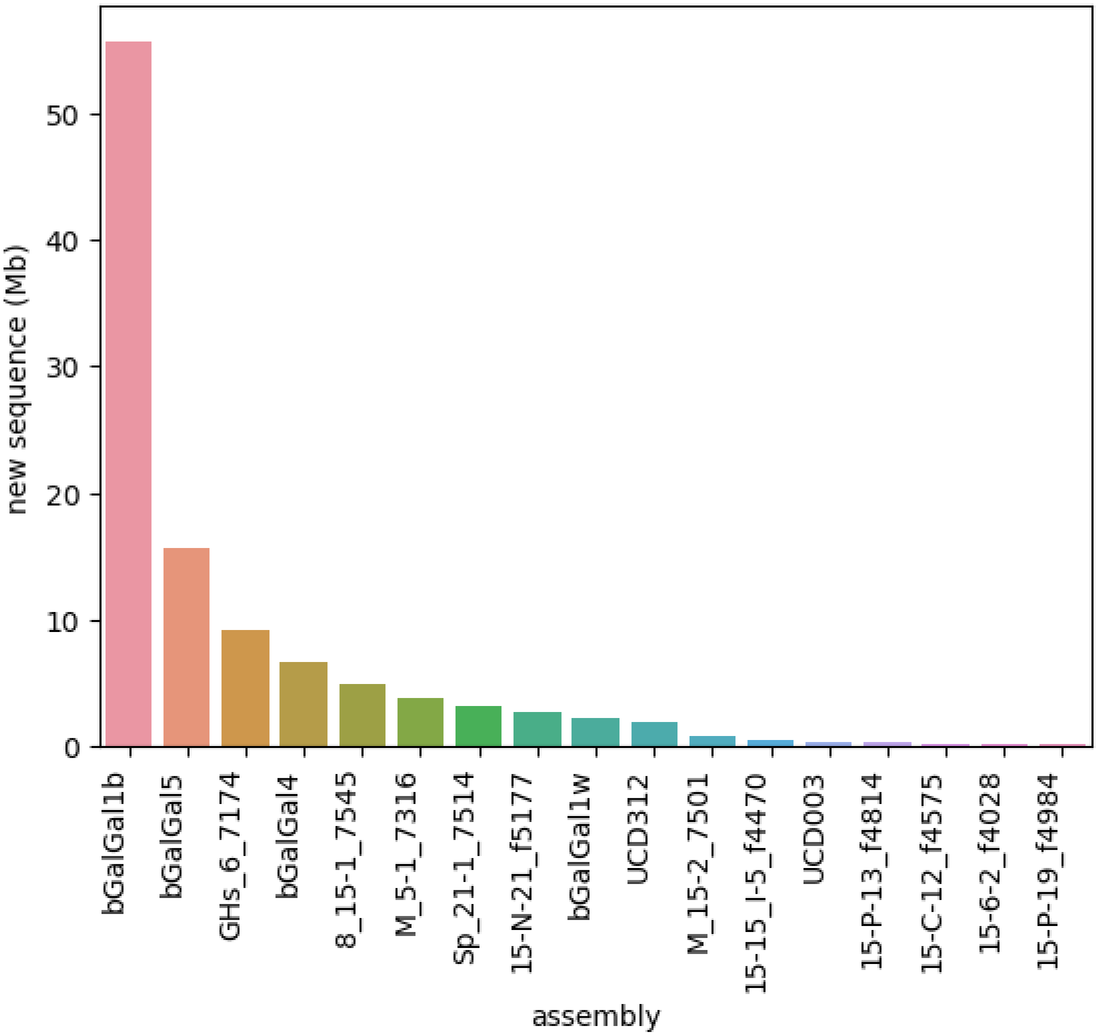

Supplementary Figure S2. Changing the parameters segment length (s), mapping percent identity (p), and minimum match length (k) affects graph structure of the PGGB graph of chr13. (a-b) 100 different parameter combinations give different values for numbers of nodes in thousands (a) and maximum degree (b). (c) While the numbers of nodes and edges differ with parameter changes, the degree (i.e., the slope of the line) remains the same. (d-e) A one-dimensional view of the graph shows that different parameter choices such as s=100000, p=99, k=350 (d) versus s=50000, p=97, k=150 (e) lead to visibly different graph structures.

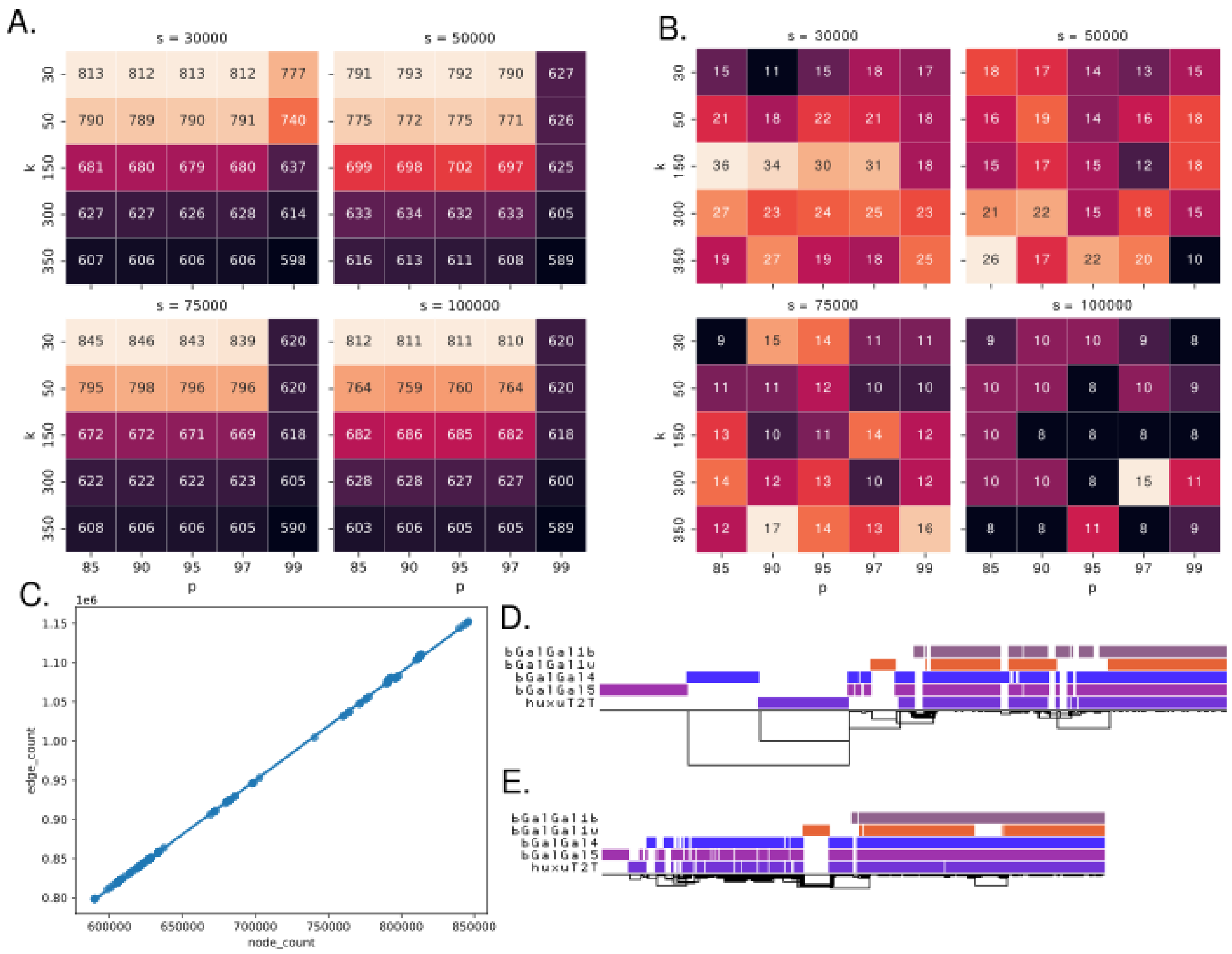

Supplementary Figure S3. A comparison of the graph structures of the first 5Mb of the 17Mb chr13 between (a) PGGB and (b) minigraph-cactus shows that the two methods produce structurally different graphs. The minigraph-cactus graph, which was built using the PacBio CLR-based bGalGal1b as a reference, is missing repetitive telomeric sequence at the beginning of the three more complete PacBio HiFi-based assemblies (bGalGal4/5 and Huxu), unlike the PGGB graph, which preserves these regions. This demonstrates a disadvantage to the reference-based approach used by minigraph-cactus.

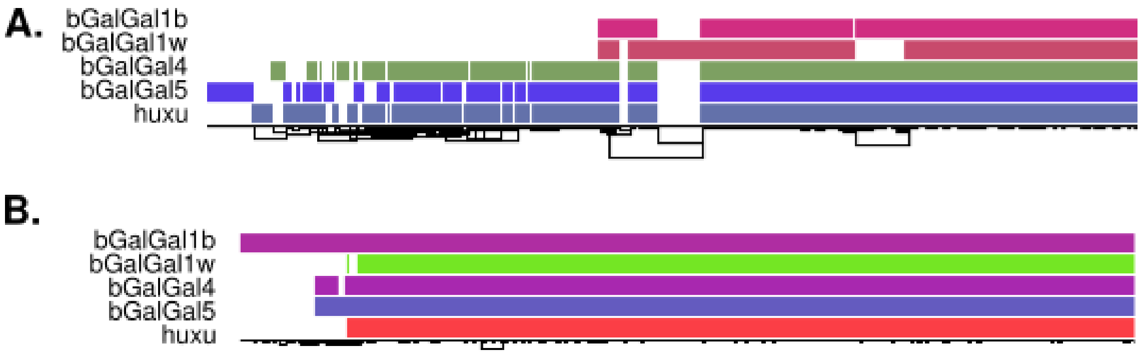

Supplementary Figure S4. Two unplaced contigs in bGalGal1w are part of the pangenome graph of the K locus, showing that bGalGal1w contains the slow feathering allele. (a) The pangenome graph of the K locus on chrZ shows two unplaced contigs from bGalGal1w are included in the graph. The contigs have a 31kb overlap with 97% identity. (b) Merging the two contigs at the overlap creates a new contig that covers the duplication present in the slow feathering allele and contains the ev21 insertion. (c) We hypothesize that these two contigs are a misassembly of the slow feathering allele, and should have been included in chrZ, which was included in bGalGal1b for the sake of a complete reference genome despite coming from the paternal layer haplotype of bGalGal1w.

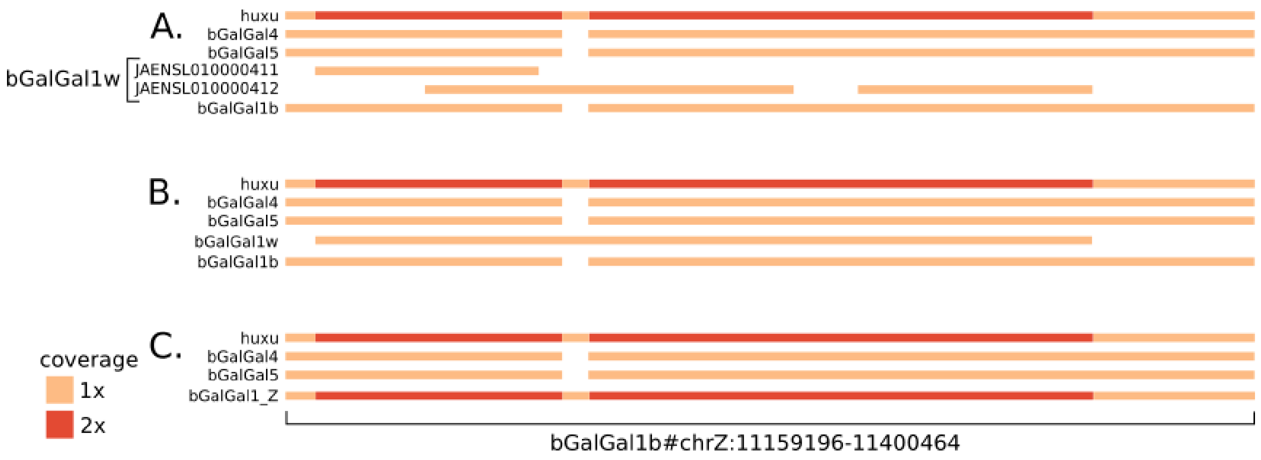

Supplementary Figure S5. Genotyping ALVEs in the pangenome. We genotyped 18 ALVEs common in commercial broilers and layers in the 30 haplotypes of the pangenome. Empty squares represent ALVE/haplotype combinations that were not able to be genotyped.

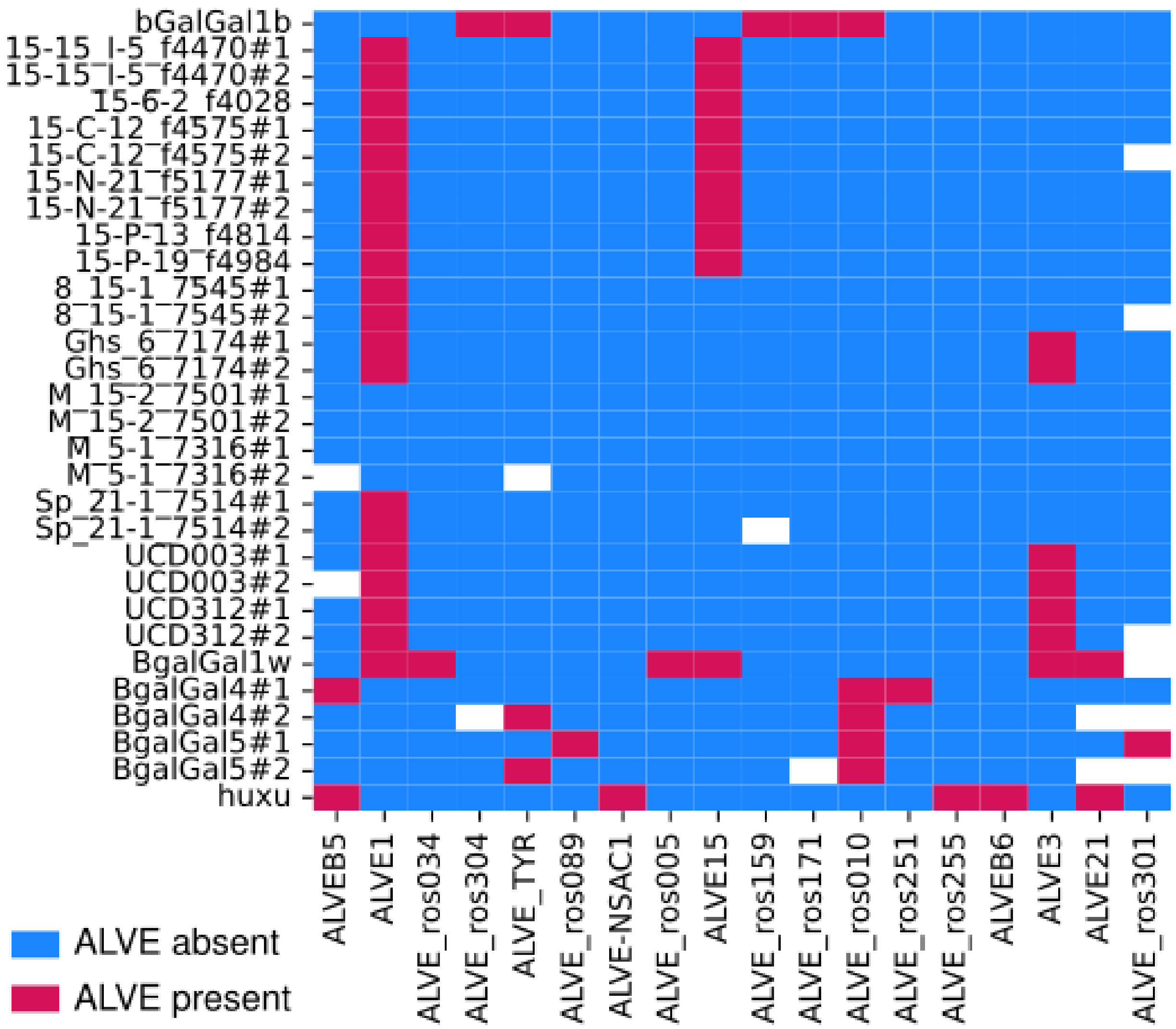

Supplementary Figure S6. Mean fractions of mapped reads containing alternate allele at putative heterozygous SNV sites. Without reference bias, this fraction would be 0.5, so larger deviations from 0.5 indicate more reference bias. For every one of the 100 short read chickens, pangenome alignment with giraffe reduces reference bias compared to linear alignment with minimap. The mean reduction across all samples is 38%. Sample information for each of these sample IDs can be found in Supplementary Table 2.

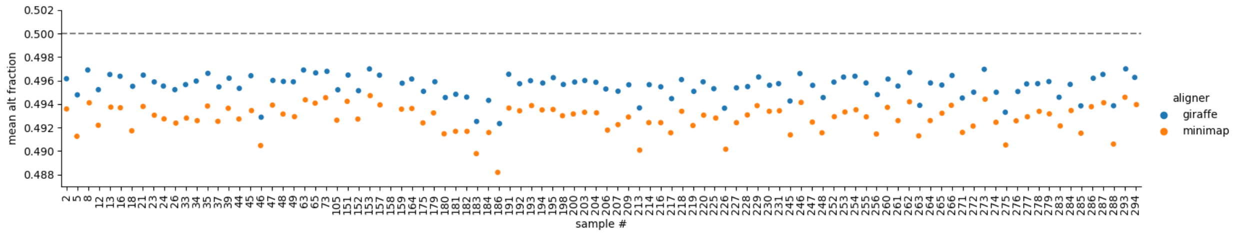

Supplementary Figure S7. Principal components analysis of genotypes for 100 short-read chickens.

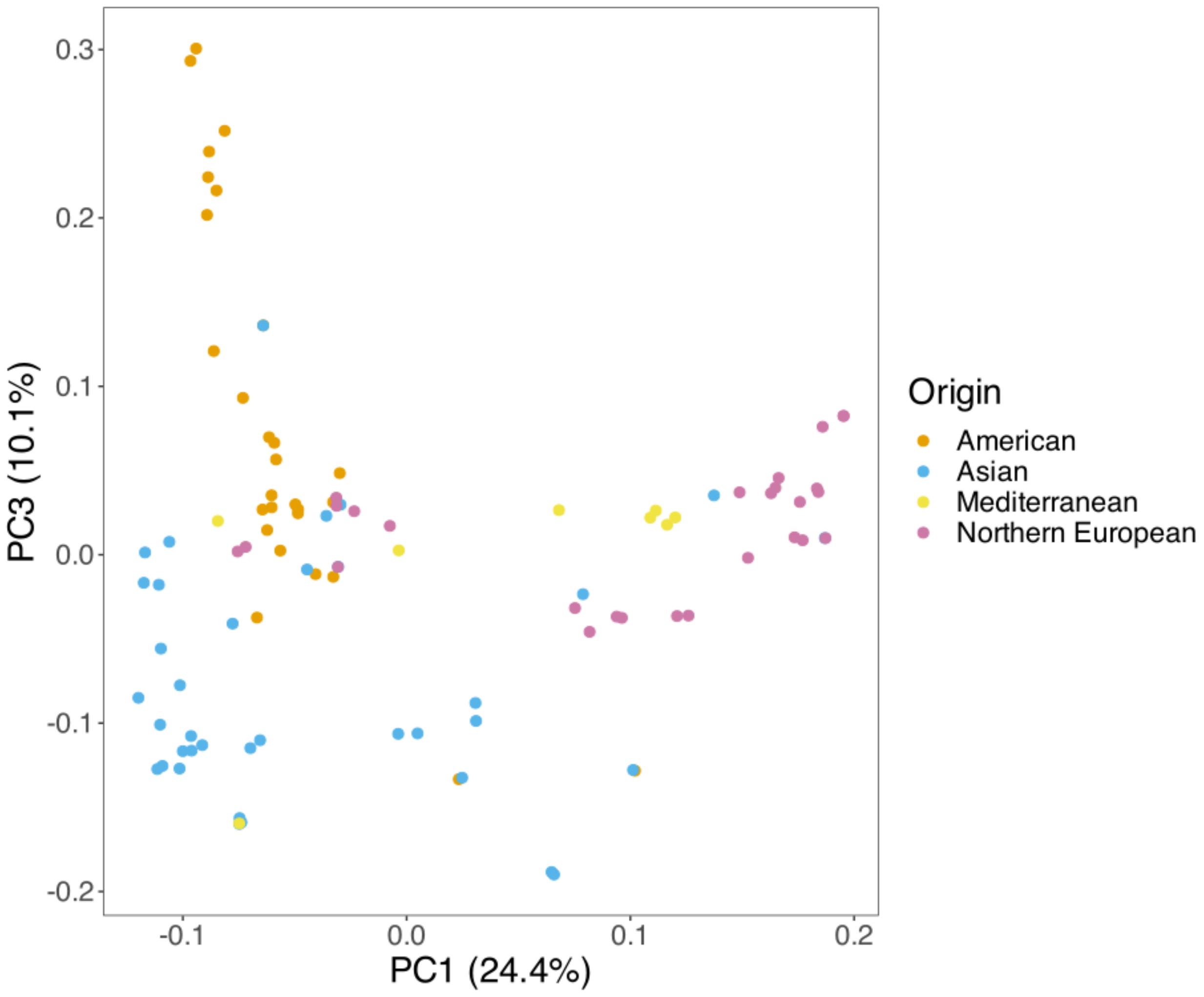

Supplement: Supplementary file 2 — Additional file 2: Figure S1. Additional sequence per sample. Figure S2. Effects of parameters on PGGB graph. Figure S3. Comparison of chr13 between PGGB and minigraph-cactus. Figure S4. Unplaced contigs at K locus. Figure S5. Genotyping ALVEs in the pangenome. Figure S6. Reference bias in giraffe vs. minimap alignments. Figure S7. PCA of short read chickens. [file 12915_2023_1758_MOESM2_ESM.pdf]
